# The Source of the Bile Pigment

**Published:** 1891-07-04

**Authors:** 


### THE SOURCE OF THE BILE PIGMENT.

The question whether there are two forms of icterus, the hæmatogenous and the hepatogenous, in the former of which there is a suppression of biliary secretion, which is consequently retained in the blood, is a point in medical pathology that has never yet been definitely solved. The hepatogenous form in which the bile is secreted by the liver, and its free passage being obstructed, is partly reabsorbed, is generally recognised, and the question is, are all forms of jaundice really and essentially hepatogenous in origin, and if so are they always due to obstruction?

In reference to the possibility of hæmotogenous jaundice, the observations of Professor Bunge are instructive: "The colouring matter of bile, bilirubin, almost certainly arises from the colouring matter of the blood, hæmatin. The following facts support this view: Biliary pigments are only found in animals whose blood contains hæmoglobin, *i.e.*, the vertebrata. The invertebrata have not, hitherto, been shown to possess them. It might be objected that this depends upon some other peculiarity of the vertebrata, as blood cells containing hæmoglobin are not the sole feature which distinguishes the vertebrata from the invertebrata. With regard to this, it is interesting to observe that the amphioxus, which has no red blood corpuscles, but which, from its whole structure, belongs to the vertebrata, forms no bile pigment. It is almost certain that there is a genetic relation between bile pigments and hæmatin. In extravasations of blood, the colouring matter of the blood disappears, and in place of it we find crystallised pigment, which Virchow named hæmatoidin, and indicated its resemblance to bile pigment. Others have subsequently proved the identity of hæmatoidin and bilirubin.*

Cases of idiopathic purpura, with accompanying jaundice, might be regarded as affording striking clinical examples of jaundice of hæmatogenous origin. The numerous and readily-formed bruises and the purpuric spots containing the altered colouring matter of extravasated red corpuscles would certainly seem to supply the source of bile pigment found in the tissues and urine.

It is very tempting to see in such facts a source of jaundice in decomposition of the blood-colouring matter taking place in the tissues or in the blood itself. But recent researches have proved, says Bunge, that there is not at present any sound basis for the conclusion that the bile pigment occurring in jaundice has any other source than the liver. He cites the experiments of Minkowski and Naunyn, who removed the liver of a goose and immediately exposed it and a healthy goose to the influence of arseniurretted hydrogen. After half an-hour the control goose evacuated urine containing biliverdin in considerable quantities, whereas the goose without the liver at first only showed a minute quantity of biliverdin, but after half an-hour hæmoglobin appeared in the urine, and the subsequently passed urine was free from bile pigment. It is, therefore, extremely probable the bile pigment in the urine in poisoning with arseniurretted hydrogen has its source in the liver.†

The communications of Mya on the origin and pathological significance of urobilin are interesting in connection with this subject. He made careful clinical observations, firstly in those cases in which there is a destruction of blood cells, such as in malaria. The most marked urobilinuria is that of malaria, and cases were examined of mild tertian ague of recent standing, in which the liver might be taken as normal. In these it progressed with the destruction of red cells, and ceased with the diminution of the specific plasmodium as the result of taking quinine.

In anæmia there exists a difference in that from loss of blood and that from hæmolysis. In the first urobilinuria is very slight. In the second form it is intense, especially in progressive pernicious anæmia and the anæmia symptomatic of cancer. In diseases of the liver it is principally in cirrhosis, chronic venous congestion, and cancer that it is most marked. In many serious hepatic affections where the epithelium is much diseased, urobilin may be scanty, and in two cases of acute yellow atrophy the jaundice was from bilirubin.‡

* Lectures on Physiological Chemistry, No. XVIII. † Ibid.

‡ Pract., Feb., 1891; La Riforma Med., No. 249, 1890.

